# Accurate, Efficient and Rigorous Numerical Analysis of 3D H-PDLC Gratings

**DOI:** 10.3390/ma13173725

**Published:** 2020-08-23

**Authors:** Jorge Francés, Sergio Bleda, Daniel Puerto, Sergi Gallego, Andrés Márquez, Cristian Neipp, Inmaculada Pascual, Augusto Beléndez

**Affiliations:** 1 Instituto Universitario de Física Aplicada a las Ciencias y las Tecnologías, Universidad de Alicante, 99, 03080 Alicante, Spain; sergio.bleda@ua.es (S.B.); dan.puerto@ua.es (D.P.); sergi.gallego@ua.es (S.G.); andres.marquez@ua.es (A.M.); cristian@ua.es (C.N.); pascual@ua.es (I.P.); a.belendez@ua.es (A.B.); 2 Departamento de Física, Ing. de Sistemas y Teoría de la Señal, Universidad de Alicante, 99, 03080 Alicante, Spain; 3 Departamento de Óptica, Farmacología y Anatomía, Universidad de Alicante, 99, 03080 Alicante, Spain

**Keywords:** H-PDLC, FDTD, diffraction efficiency, LD director distribution, Frank elastic free energy

## Abstract

This work presents recent results derived from the rigorous modelling of holographic polymer-dispersed liquid crystal (H-PDLC) gratings. More precisely, the diffractive properties of transmission gratings are the focus of this research. This work extends previous analysis performed by the authors but includes new features and approaches. More precisely, full 3D numerical modelling was carried out in all analyses. Each H-PDLC sample was generated randomly by a set of ellipsoid geometry-based LC droplets. The liquid crystal (LC) director inside each droplet was computed by the minimisation of the Frank elastic free energy as a function of the applied electric field. The analysis carried out considered the effects of Frank elastic constants $K_{11}$, $K_{22}$ and $K_{33}$; the anchoring strength $W_{0}$; and even the saddle-splay constant $K_{24}$. The external electric field induced an orientation of the LC director, modifying the optical anisotropy of the optical media. This effect was analysed using the 3D split-field finite-difference time-domain (SF-FDTD) method. In order to reduce the computational costs due to a full 3D tensorial analysis, a highly optimised method for high-performance computing solutions (HPC) was developed. The influences of the anchoring and voltage on the diffraction efficiencies were investigated, showing the potential of this approach.

## 1. Introduction

Many applications, such as diffraction lenses, optical data storage, image capture devices and solar cell applications, amongst others, are related to holographic polymer dispersed liquid crystals (H-PDLCs). The combination of a light-sensitive monomer and liquid crystal (LC) mixture and their exposition to an interference pattern sets up H-PDLC devices. The highly irradiated zones produce a lack of monomer due to polymerisation. This effect provokes an increase of the monomer concentration in the illuminated areas, thereby producing a periodic pattern. The grating is based on a set of small LC droplets surrounded by a polymer. Many researchers have focused their efforts into improving the diffraction efficiency or the angular selectivity [1,2] with lower driving voltages [3]. It is also worth noting novel applications of H-PDLC, e.g., H-PDLC with variable period [4] and H-PDLC gratings used for autostereoscopic display [5,6], amongst others. In order to achieve these goals, many theoretical and numerical approaches have been developed, e.g., the application of Montemezzani’s [7] coupled waves theory for gratings to shaped-droplets [8], models of droplet axis reorientation [9] and the effective medium theory [10] applied to the analysis of reflective H-PDLC. Kubitskiy et al. [11] have contributed significantly to the application of FDTD to H-PDLC. It is worth noting the analysis performed in [12], wherein the LC director distribution was computed by means of the application of the Monte-Carlo method, and a two-dimensional FDTD scheme was used for obtaining the light propagation along the grating. Wang et al. [13] and Gui et al. [14] also contributed to the analysis of H-PDLC using FDTD method for one-dimensionally periodic structures [13], and H-PDLC with embedded silver nanoparticles [14], respectively. It is also interesting to mention contributions more focused on the analysis of the properties of spatial light modulators based on a nematic zero-twist liquid-crystal (NLC); e.g., [15] computed the LC director by minimising the electric and elastic-free energies for one-dimensionally periodic structures, [16] analysed crosstalk in liquid spatial light modulators following a rigorous study of the LC director distribution as well and [17,18] followed a similar approach, also considering three-dimensional simulations and FDTD numerical method. The authors applied a two-dimensional split-field (SF) FDTD method to the analysis of ellipsoidal droplets in H-PDLC gratings with random properties [19]. In this contribution previously mentioned, the Monte-Carlo method was applied to random ellipsoidal droplets for obtaining the LC director distribution. Many parameters related to the physical packing of the droplets were considered, e.g., the density packing, grating period, upper and lower limits of the ellipsoid size and initial LC director state. The generation of a random particle and its packing is not a straightforward issue. Actually, granular packing studies are relevant to physicists, biologists and engineers [20,21]. The viscosities of particle suspensions are affected by the packing fraction [22]. Collisional processes in astronomy are also analysed numerically by creating a set of fragments with different shapes (cubes, spheres, etc.) [23]. Here, random ellipsoids were generated and allocated inside the polymerised region of the H-PDLC. It is worth noting that the packing limit for spheres has been well established at a density of Ω≈0.64 [21,24]. However, in this work lower limits are considered in order to be closer to non-homogeneous H-PDLC droplet configurations, thereby triggering light scattering.

In this work, a full tensorial three-dimensional numerical analysis was carried out using the SF-FDTD method. Maxwell’s curl equations govern the propagation of light waves. FDTD simulations solve Maxwell’s equations by approximating both time and spatial derivatives by means of central differences [25,26] in a discrete grid. The FDTD method is a powerful approach since it provides information of the electromagnetic waves as a function of time and space. SF-FDTD is a specified version of the standard FDTD method for the analysis of electromagnetic waves with an oblique angle of incidence along with periodic media. This method has been recently used in many applications wherein diffractive elements are analysed [26,27,28]. The analysis of anisotropic media implies considering the tensorial behaviour of the permittivity tensor ϵ, and increasing the complexity of the method and the computational resources as well [27,28]. The finite difference method has a computational cost that grows exponentially with the grid size. Thus, considering anisotropic media and 3D full simulations can be demanding for volume gratings or periodic structures with dimensions larger than the work wavelength. Here, some acceleration strategies have been considered in order to increase the performance, thereby reducing the running time per simulations, making this approach competitive. SF-FDTD implementation was completely developed in C++ by the authors. This complete control on the software has permitted us to include new features such as parallel computing based on GPUs and CUDA. Interested readers can find more details related to the implementation of the method in [26,27,28,29,30,31] regarding the computational acceleration through parallel and GPU computing. It is worth noting a free implementation of the method (WOLFSIM: https://sourceforge.net/projects/wolfsim/). The details of this implementation are provided in the outstanding works of Oh and Escuti [26] and Miskiewicz et al. [28].

In order to simulate H-PDLC structures, some considerations must be clarified. H-PDLC devices are based on a set of droplets filled with LC material surrounded by a polymer. The polymer is considered isotropic, so the $\epsilon_{ii}$ components of the tensor are well known in this case. However, the refractive index perceived by input light in the different LC droplets depends on the external control voltage and the induced orientation of the director n inside the LC droplet. Therefore, it is necessary to estimate the director’s orientation as a function of the geometry, the LC characteristics and the external control voltage. The minimisation of the total free energy [16,32] was the formalism chosen for solving this aspect. The minimisation of the free energy considers the contributions of the deformation, surface and electric field [30]; i.e., the solution considers the influence of $K_{11},K_{22},K_{33},K_{24}$ elastic constants, anchoring strength and external field. The impact of the elastic constants has been widely analysed. The influence of the saddle splay constant $K_{24}$ is not usually considered for LC-based devices such as spatial modulators. However, it has been reported that the $K_{24}$ term has a direct influence on the droplet structure, even in the limit of the zero anchoring strength for H-PDLC devices [33,34] when the surface-to-volume ratio is high [32]. On the other hand, the influence of the $K_{13}$ parameter has been neglected in this work since it has been demonstrated that its anchoring strength at the LC interface is negligible [33]. Once the director’s orientation is obtained, the components of the dielectric tensor of the LC material are computed easily through the well-known dependence between the LC material and the dielectric tensor ϵ via n [16].

The scheme here proposed was applied to an H-PDLC sample with random droplets. After the sample is defined, the input parameters regarding physical characteristics of the LC, polymer or external voltage are considered for the minimisation of the free energy. The estimated director distribution permits to obtain the different components of the permittivity tensor, which is considered for the numerical FDTD simulation that provides the electromagnetic field in three dimensions. The analysis carried out includes different anchoring strengths, and different values for the external field applied. The influences on the diffraction efficiency of these parameters is analysed as a function of the incidence angle of the input light.

The strategy considered differs significantly from our previous work [19]. Here, three-dimensional simulations were carried out, which were done to analyse two-dimensionally periodic structures instead of only one-dimensional periodic structures. Moreover, a rigorous formalism based on the minimisation of the free energy was used for determining the director distribution of the LC droplets, instead of considering statistical approaches such as the one considered in [19]. This solution brings about the opportunity of modelling accurately LC-based devices in a sub-micron regime, taking into account the physical parameters of the media considered, and the external control voltage or the LC constants, for instance. The model brings about the possibility of performing an inverse procedure in order to fit physical parameters while taking into account experimental data, or predicting the behaviour of an H-PDLC sample before its production. The results herein presented show the potential of the setup since the scheme provides the diffraction efficiency as a function of the external field amplitude for a different angle of incidence and anchoring conditions.

## 2. Physical Model

The analysis carried out in this work was based on three main steps. Firstly, the H-PDLC sample is generated randomly. In this step, each LC droplet is created with arbitrary characteristics (considering a set of constraints) and allocated inside the grating following the input parameters provided (period, thickness, LC refractive indices, etc.). Once the sample is finally generated, the next step is to compute the LC director for each droplet. Note that the whole sample is spatially discretised; thus, each LC droplet can be composed of many cells in which an LC director and voltage are defined. After the LC director is obtained, the permittivity tensor is calculated from the LC director and provided to the SF-FDTD method for estimating the light propagation along the H-PDLC. In the following subsection, each phase is detailed in-depth. While it would be natural to start detailing each step from the sample creation up to the SF-FDTD formulation, the authors consider that it is more appropriate to start detailing the numerical method, since it presents the key parameters of this work and also helps to define the problem spatially.

### 2.1. Numerical Solution of the Optical Field

**Figure 1:**
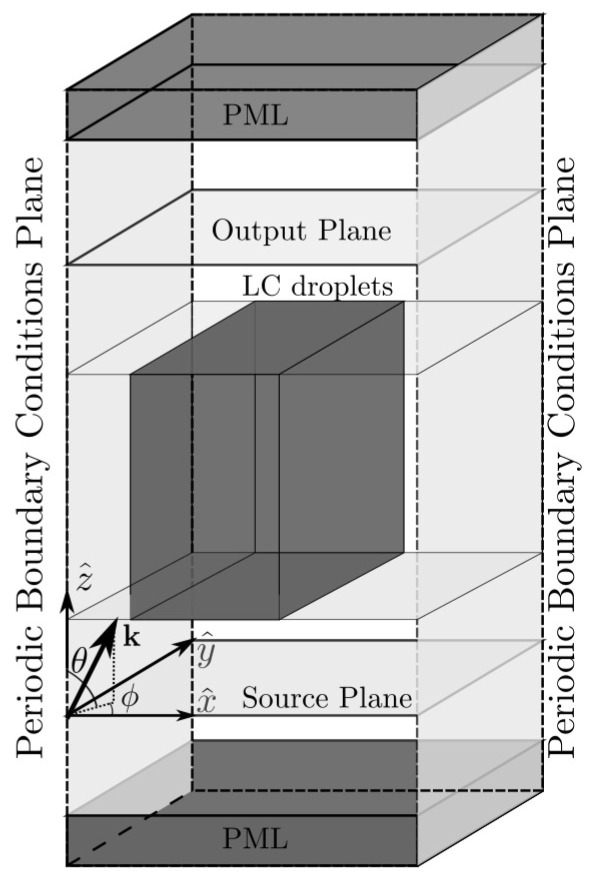
Scheme of the SF-FDTD simulation.

The model proposed in here is based on a three-dimensional H-PDLC structure. The H-PDLC medium is discretised in order to be able to determine the LC director of each cell of the grid. This mesh is used for solving Maxwell’s equations using the SF-FDTD method. Figure 1 shows the SF-FDTD scheme considered in this work. In our model, incident light propagates along the $\hat{z}$ axis forming an oblique angle of incidence θ with respect to the $\hat{x}$ axis. Here, linear vertical light along $\hat{y}$-axis is considered. The incidence plane $\hat{x}\hat{z}$ contains both the wavenumber k of the input light and the normal to the H-PDLC sample. Periodic boundary conditions (PBC) are applied in both $\hat{y}$ and $\hat{x}$ axes. For the sake of better understanding, an H-PDCL grating with a spatial variation (grating vector) along the $\hat{x}$ axis is considered. That is to say that the droplets are distributed homogeneously along the $\hat{z}$ direction. Indeed, it may initially appear that two-dimensional analysis is carried on, but three-dimensional information related to the LC director and the anisotropy of the medium is completely incorporated in our analysis. Perfectly matched layers (PML) are considered at the upper and bottom boundaries in $\hat{z}$ axis. PML boundaries are used for avoiding artificial reflections in non periodic boundaries, thereby simulating propagation in free space along the $\hat{z}$ axis.

**Figure 2:**
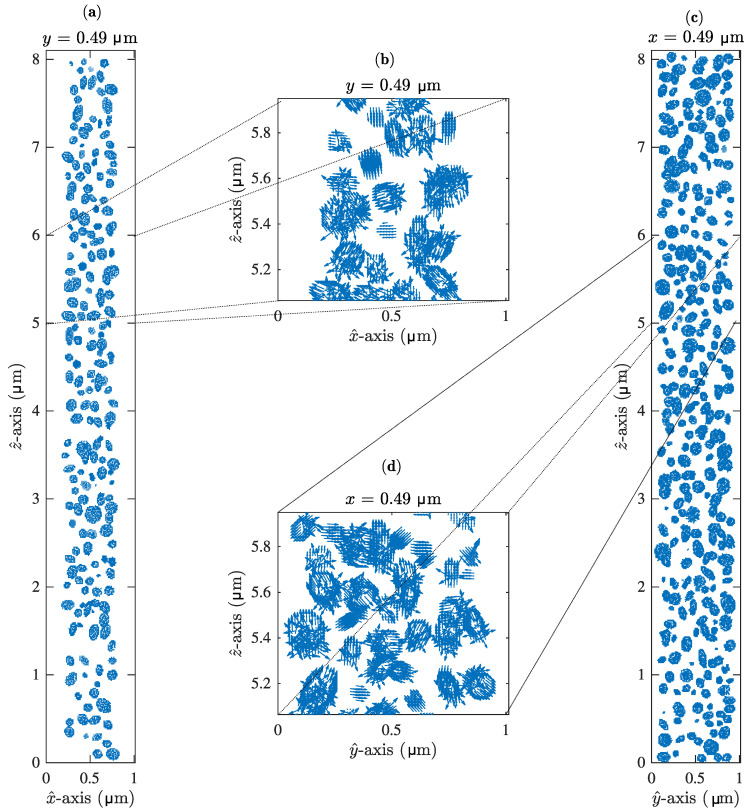
Representation of the initial random LC director through some slices of the H-PDCLC sample generated, here the anchoring strength is $W_{0}$ = 10${}^{-5}$ J/m${}^{2}$ and Φ=0 V: (**a**) xz plane. (**b**) Zoom in of a region in the xz plane. (**c**) yz plane. (**d**) Zoom in of a region in the yz plane.

Figure 2 shows different slices of the three-dimensional sample considered. More precisely, Figure 2 represents the sample and the LC droplet area shown in Figure 1, zoomed in order to be able to perceive the different LC droplets of the H-PDLC sample generated. This figure shows different planes in order to illustrate the symmetry of the problem.

SF-FDTD method updates the electromagnetic field as a function of both time and space by employing a set of new transformed variables [28,35]:

(1) $$\begin{array}{ccc} P & = & Ee^{j(k_{x}x+k_{y}y)}, \end{array}$$

(2) $$\begin{array}{ccc} Q & = & c\mu_{0}He^{j(k_{x}x+k_{y}y)}. \end{array}$$

SF-FDTD considers the propagation of planewaves propagating in the k direction defined as

(3) $$k=k_{x}\hat{x}+k_{y}\hat{y}+k_{z}\hat{z}=\frac{\omega }{c}\left(\sin\theta \cos\phi \hat{x}+\sin\theta \sin\phi \hat{y}+\cos\theta \hat{z}\right),$$

where θ is the polar angle from the $\hat{z}$ axis, and ϕ is the azimuthal angle from the $\hat{y}$ axis. *c* is the speed of light waves on vacuum and $\mu_{0}$ is the magnetic permeability constant. Note that nonmagnetic materials are assumed to have the permeability tensor μ unity. An interested reader (in SF-FDTD formulation) can consult some essential references in the literature such as [35], the contributions of the research group of Dr. Escuti [26,27,28] and some interesting works related to the consideration of new addons in SF-FDTD by Shahmansouri and Rashidian [36]. The authors have also contributed in the development of this method in some works [29,37,38].

### 2.2. Director Distribution of the LC Droplets

For computing the light behaviour in a H-PDLC the orientation of the LC director must be well defined. LC director is defined as a unit vector pointing into the (average) direction of the long molecule axis n [16]. For estimating the LC director distribution under the influence of an electric field E, we follow the approach described in [16,32,33]. The aim of this procedure is to find a director distribution that minimises the total free energy. Free energy is usually divided in different contributions, i.e., the homogeneous free energy $f_{O}$, elastic $f_{e}$, interfacial $f_{s}$ and field $f_{f}$. Due to the fact that this study is focused on the analysis of relatively large droplets, the term $f_{O}$ can be neglected, since it is just a temperature-dependent parameter [33]. Frank elastic free energy density is related to the inhomogeneous part of the nematic free energy density $f_{e}$.

(4) $$f_{e}\left(r\right)=\frac{1}{2}K_{11}{\left(\cdot n\right)}^{2}+\frac{1}{2}K_{22}{\left(n\cdot \nabla \times n\right)}^{2}+\frac{1}{2}K_{33}{\left(n\times \nabla \times n\right)}^{2}-\frac{1}{2}K_{24}\nabla \cdot \left(n\left(\nabla \cdot n\right)+n\times \nabla \times n\right).$$

The interaction of the LC with the surrounding medium can be described as

(5) $$f_{s}\left(r\right)=\left(1-{\left(n\cdot e_{r}\right)}^{2}\right)\frac{W_{0}}{2}\delta \left(r-R\right),$$

where $e_{r}$ is the preferred anchoring direction on the droplet surface and $W_{0}$ the anchoring strength [33]. The magnitude of the anchoring strength and its influence on the director n depend on the elastic constants, for instance. Usually, weak and strong anchoring strengths are considered in their upper and bottom limits. Weak anchoring can be established near to 10${}^{-5}$ J/m${}^{2}$ (page 165 in [32]), whereas strong anchoring in the same material can be expected around a few orders of magnitude higher. However, these two extreme boundary conditions are usually applied considering $W_{0}$ almost 0 for weak anchoring and *∞* for strong anchoring. Here, a wide range covering several orders of magnitude for $W_{0}$ has been considered in order to identify the transition between different anchoring behaviours.

The vector R in Equation (5) represents the points on the droplet surface. Here, the interaction with an external field is described by [16]:

(6) $$f_{f}\left(r\right)=-\frac{1}{2}\epsilon E\cdot E,$$

E being the electric field inside the LC. The dielectric tensor ϵ of the LC material is related to the director n by the following relationship:

(7) $$\epsilon_{ij}=\epsilon_{⊥}\delta_{ij}+\left(\epsilon_{\|}-\epsilon_{⊥}\right)n_{i}n_{j},$$

with $\epsilon_{\|}$ and $\epsilon_{⊥}$ being the relative permeability for LC aligned parallel and perpendicular to the electric field direction. A complete solution of the electric field can be obtained using Maxwell equations at the same time that the free energy is minimised. The minimisation of the total free energy is performed by solving the Euler–Lagrange differential equations

(8) $$-F_{i}=\frac{\partial f}{\partial n_{i}}-\sum_{j=1}^{3}\frac{\partial }{\partial x_{j}}\left(\frac{\partial f}{\partial n_{i,j}}\right)=0\;\mathrm{for}\;i\in \left\{1,2,3\right\},$$

where *f* is the linear contribution of the energies defined previously

(9) $$\int fdV=\int (f_{O}+f_{e}+f_{s}+f_{f})dV\to \mathrm{minimal}.$$

Equation (8) was solved by numerically approximating the derivatives with central finite differences. The procedure was fully detailed in [16] in Equation (7), where the electrostatic potential Φ and n were iteratively updated. Note that the electric field E is related to the potential Φ through the Gauss’s law. The coupled non linear partial differential equations were solved numerically on a grid that includes the LC droplets and the polymer altogether. A was mentioned in the previous section, periodic boundary conditions were applied in $\hat{x}$ and $\hat{y}$ axis, whereas along $\hat{z}$ axis, the given voltages applied on the electrodes of the H-PDLC sample constrained the electric potential. As a starting guess, the potential was defined as a linear ramp along $\hat{z}$ between the electrodes, and for n the LC distribution was initially totally random. More specifically, for each droplet $e_{r}$ was defined parallel to its larger semi-major axis, which is arbitrary as well. This condition is fulfilled on the surface of each droplet that is randomly placed and oriented inside the H-PDLC. The LC director inside each droplet, which is surrounded by the surface previously defined, is totally random, and initially, does not receive any influence from the droplet surface. However, depending on the anchoring strength, the LC director inside the droplet will be oriented, taking into consideration the surface constraints.

### 2.3. Random H-PDLC Sample Created by Packing Ellipsoids

The packing algorithm tries iteratively to fit new droplets of 40–100 nm size inside the grating [12]. A user can modify the length of the polymerised area. For us, a grating with the 50% of the period polymerised was considered. The surface of each droplet is identified and labelled in order to be designed with a preferred direction of the LC director ($e_{r}$). This unit vector is parallel to the largest semi-major axis of the ellipsoid. This direction is entirely arbitrary since the size of the three-axis of the ellipsoid, and its rotation are also arbitrary. This process is performed iteratively till the desired packing density is fulfilled. Note that this process can be very long and time-consuming, since the higher the packing density, the lower the chance of finding a proper spot for new LC droplets. Once the sample is completed, a spatial grid with LC droplets is defined. The grid identifies the position of each LC droplet and the surface of them as well. The parameters used for the simulations are given in Table 1. LC properties were extracted from [16] and the value of $K_{24}$ was considered closer to $K_{11}=K_{22}=K_{33}$ following the results given in [33]. The source was 633 nm polarized at 90${}^{\circ }$ light. The incidence angle θ was varied between $-30^{\circ }$ and 30${}^{\circ }$, whereas ϕ = 0${}^{\circ }$ in all cases.

The grid size for the H-PDLC structure was composed of an arrangement of 640 × 80 × 80 cells along the $\hat{z}$, $\hat{x}$ and $\hat{y}$ axes, respectively. Figure 2 shows some slices of the LC director for the three-dimensional domain simulated. The configuration shown in Figure 2 represents the initial state for the different procedures detailed previously, i.e., minimisation of the total free energy, and numerical simulation of the optical fields.

## 3. Results

**Figure 3:**
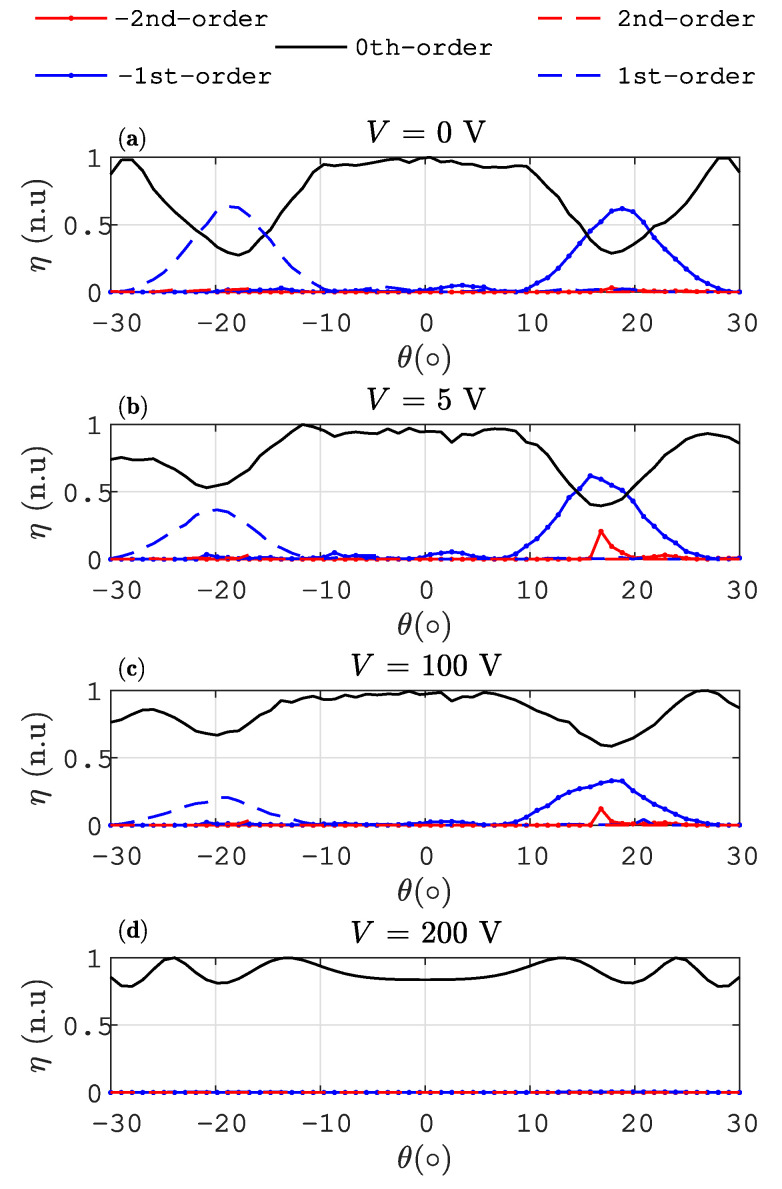
Diffraction efficiency for the orders ± 1st and 0th as a function of the angle of incidence $\theta_{0}$ (**a**) Φ = 0 V. (**b**) Φ = 5 V. (**c**) Φ = 100 V. (**d**) Φ = 200 V.

Figure 3 shows the diffraction efficiency η as a function of the incidence angle $\theta_{0}$ for different external control voltages (Φ). It can be seen how the diffraction efficiency for ± 1st orders is dramatically reduced as the voltage is increased. For the highest voltage, the LC director is aligned towards the normal layer direction, thereby producing a matching between the LC ordinary refractive index and the one of the polymer. In this extreme situation (Φ = 200 V), the grating almost disappears, and a homogeneous dielectric layer is perceived by input light. It is worth noting that the magnitude of the external voltage is usually very high in PDLC structures [32]. Some differences between the ±1st-orders can be produced by the normalisation (|n| = 1) of the LC director during the time relaxation procedure. This normalisation can produce a preferred orientation of the LC director, inducing a small asymmetry of the grating.

**Figure 4:**
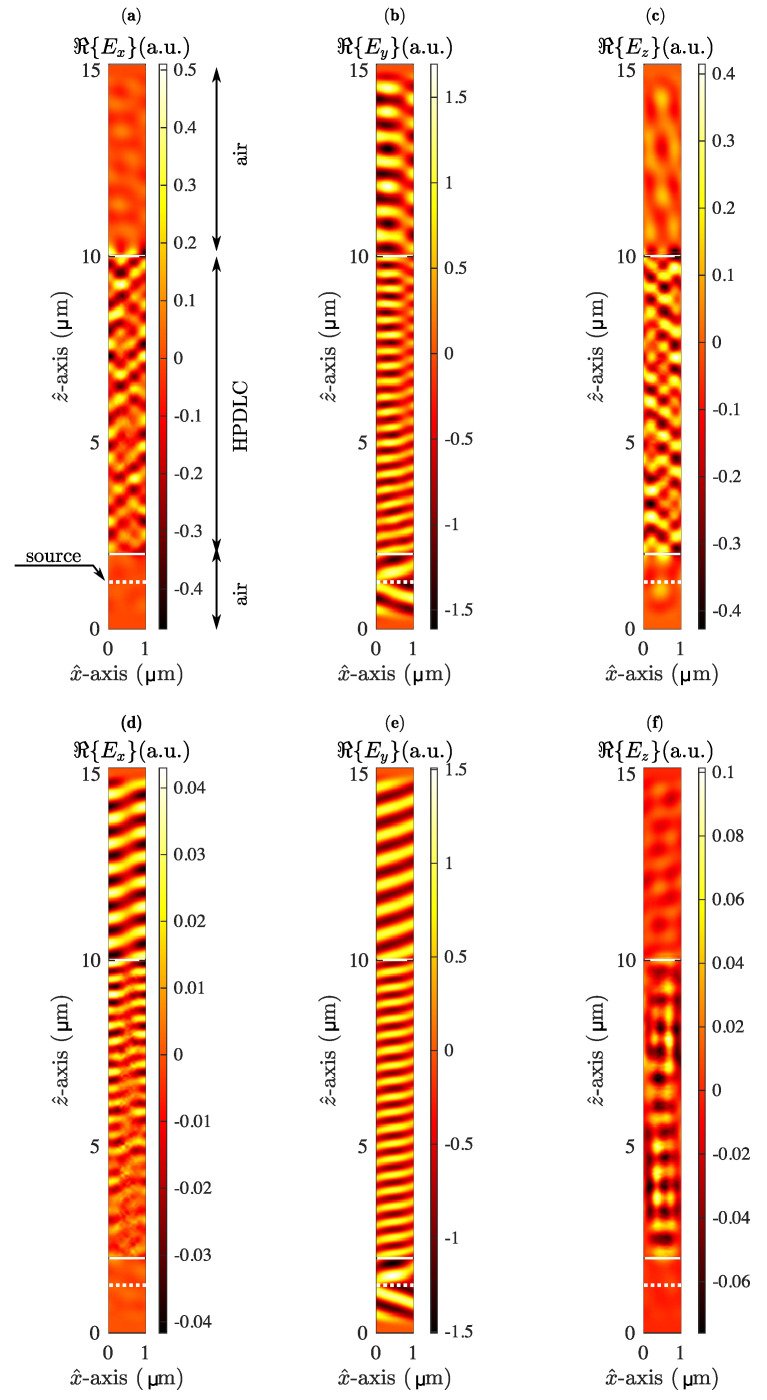
Representation of the xz plane slice of $ℜ\{E_{i}\}$, with i=x,*y* and *z*. In all cases *y* = 0.49 μm and $W_{0}$ = 10${}^{-5}$ J/m${}^{2}$. (**a**), (**b**) and (**c**) show the *x*, *y* and *z* components of the electrical field for Φ=0 V, respectively. (**d**), (**e**) and (**f**) show the *x*, *y* and *z* components of the electrical field for Φ=200 V, respectively.

In order to demonstrate the potential of this approach, the electric field for the three spatial components is represented in Figure 4 in the Bragg’s angle (18.8${}^{\circ }$). In all cases the input light is linear vertical polarisation ($E_{y}$) with an oblique angle of incidence. Since for the input voltage Φ = 0 V the grating is present, and the Bragg condition is achieved, the mixture of the 0th and 1st order can be identified in transmission (see Figure 4b). Due to the inner anisotropy of the LC some light is transferred into the $\hat{x}$ and $\hat{z}$ components, detailed in Figure 4a,c. Figure 4d–f represents the same set of fields, but in this case for the maximum control voltage Φ=200 V. Here, the spatial variation of the refractive index is very small; thus, the input light remains almost intact. Note that the amplitudes of the $\hat{x}$ (Figure 4d) and $\hat{z}$ (Figure 4f) light contributions are up to 10 times lower compared to the amplitudes shown in Figure 4a,c, respectively. It is interesting to clarify that all FDTD simulations were configured in order to ensure the steady-state performing more than 15,000 time-steps. This setup provides the behaviour of the light after being inside the grating for more than 30 ps. This time interval can be reduced slightly for an oblique angle of incidence due to the Courant condition that ensures FDTD stability. However, the authors have corroborated empirically that the steady-state has been reached in all situations.

**Figure 5:**
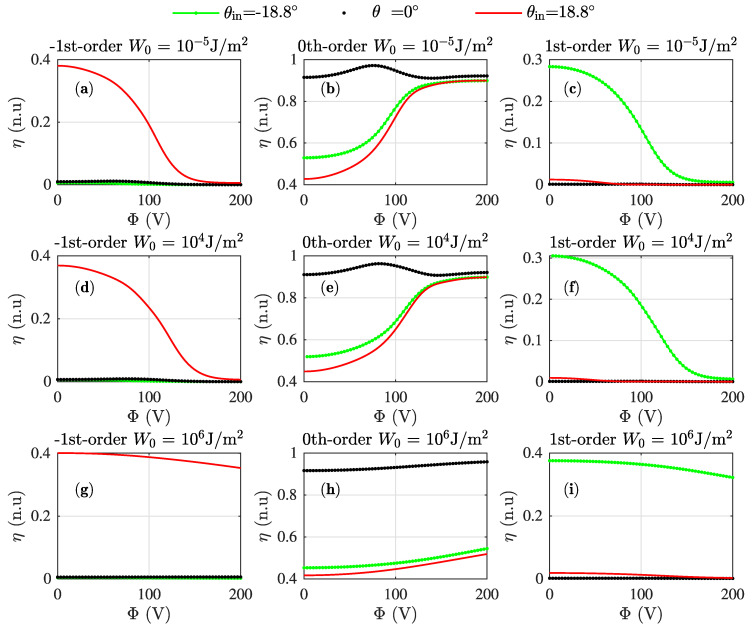
Diffraction efficiencies of ±1st-order and zeroth-order as a function of the control voltage with anchoring strength $W_{0}$ = 10${}^{-5}$ J/m${}^{2}$ and different incidence angles:(**a**) -1st-order, $W_{0}$ = 10${}^{-5}$ J/m${}^{2}$. (**b**) 0th-order, $W_{0}$ = 10${}^{-5}$ J/m${}^{2}$. (**c**) 1st-order, $W_{0}$ = 10${}^{-5}$ J/m${}^{2}$. (**d**) -1st-order, $W_{0}$= 10${}^{4}$ J/m${}^{2}$. (**e**) 0th-order, $W_{0}$ = 10${}^{4}$ J/m${}^{2}$. (**f**) 1st-order, $W_{0}$ = 10${}^{4}$ J/m${}^{2}$.(**g**) -1st-order, $W_{0}$ = 10${}^{6}$ J/m${}^{2}$. (**h**) 0th-order, $W_{0}$ = 10${}^{6}$ J/m${}^{2}$. (**i**) 1st-order, $W_{0}$ = 10${}^{6}$ J/m${}^{2}$.

**Figure 6:**
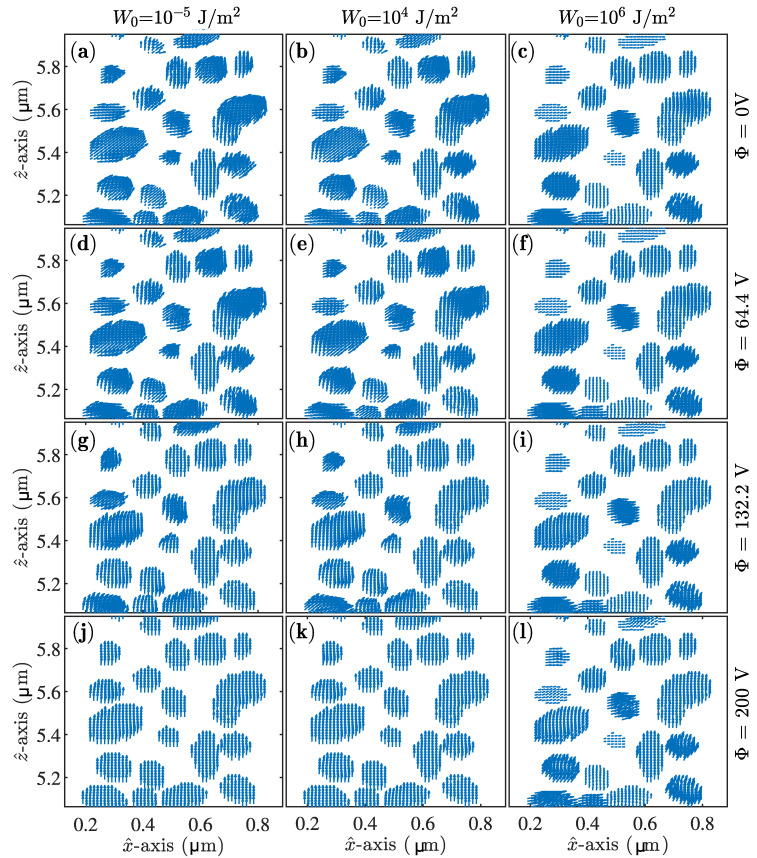
LC director representation for the $\hat{x}\hat{z}$ plane at *y* = 0.49 μm. (**a**) Φ = 0 V and $W_{0}$ = 10${}^{-5}$ J/m${}^{2}$. (**b**) Φ = 0 V and $W_{0}$ = 10${}^{4}$ J/m${}^{2}$. (**c**) Φ = 0 V and $W_{0}$ = 10${}^{6}$ J/m${}^{2}$. (**d**) Φ = 64.4 V and $W_{0}$ = 10${}^{-5}$ J/m${}^{2}$. (**e**) Φ = 64.4 V and $W_{0}$ = 10${}^{4}$ J/m${}^{2}$. (**f**) Φ = 64.4 V and $W_{0}$ = 10${}^{6}$ J/m${}^{2}$. (**g**) Φ = 132.2 V and $W_{0}$ = 10${}^{-5}$ J/m${}^{2}$. (**h**) Φ = 132.2 V and $W_{0}$ = 10${}^{4}$ J/m${}^{2}$. (**i**) Φ = 132.2 V and $W_{0}$ = 10${}^{6}$ J/m${}^{2}$. (**j**) Φ = 200 V and $W_{0}$ = 10${}^{-5}$ J/m${}^{2}$. (**k**) Φ = 200 V and $W_{0}$ = 10${}^{4}$ J/m${}^{2}$. (**l**) Φ = 200 V and $W_{0}$ = 10${}^{6}$ J/m${}^{2}$.

Figure 5 shows the variation of the -1st-order (Figure 5a,d,g), 0th-order (Figure 5b,e,h) and 1st-order (Figure 5c,f,i). The diffraction efficiency is represented as a function of the external voltage for normal incidence and Bragg angles ± 18.8${}^{\circ }$. The influence of the anchoring strength was also analysed by varying this parameter for each row of graphs. Actually, the first row of graphs shows the results for a weak anchoring situation ($W_{0}$ = 10${}^{-5}$ J/m${}^{2}$). The anchoring is increased in each row, from up to bottom in Figure 5. It can be seen how the impact of the anchoring strength becomes evident for $W_{0}\approx 10^{4}$ J/m${}^{2}$ approximately. The higher the anchoring strength is, the lower the effect of higher control voltages is on the grating, thereby maintaining higher diffraction efficiencies even for the highest control voltage considered. These results demonstrate the necessity of applying huge voltages in H-PDLC devices, since it is well known that the surface and anchoring effects on the droplets are quite high [32]. For providing more evidence of this behaviour, some LC distributions as a function of the anchoring and the external control voltages are summarised in Figure 6. More specifically, the LC director for different anchoring strengths is shown in column order, whereas the voltage is increased for each row of graphs. The LC distribution for Figure 6a,d,g,j, with $W_{0}$ = 10${}^{-5}$ J/m${}^{2}$, shows clearly how the LC director of the droplets gets oriented parallel to the $\hat{z}$-axis reasonably well even for Φ = 132.2 V. Some differences can be identified in the surface area in the bottom droplets shown in Figure 6g that are totally aligned in Figure 6j. It is interesting to address that the LC director is almost not voltage influenced in the lower-mid voltage range since the parts of Figure 6a,d are very similar.

Figure 6b,e,h,k covers the LC director distribution for $W_{0}$ = 10${}^{4}$ J/m${}^{2}$. For mid-range voltages (2nd and 3rd rows of graphs) the differences are noticeable, since the anchoring was increased, and the LC director, on boundaries, tended to be more static, even for high voltages. However, Figure 6h,k shows that the increase of the control voltage is enough for forcing the alignment of the LC director. Finally, Figure 6c,f,i,l covers the strong anchoring case ($W_{0}$ = 10${}^{6}$ J/m${}^{2}$). If Figure 6c,f is compared with those related to weaker anchoring, it can be seen how the surface boundaries influence in a great manner the arrangement of the LC director in the low–mid range of the external voltage. Figure 6l covers the case for both the highest control voltage and strongest anchoring. Here, some differences can be seen in the droplet allocated in the centre of this graph and some in the bottom. The LC director allocated inside these droplets shows a good vertical alignment induced by the influence of external voltage. However, the strong anchoring forces boundaries to remain almost intact compared to Figure 6c,f,i.

## 4. Conclusions

This work shows the results derived from a rigorous numerical analysis of H-PDLC. H-PDLC was created with random ellipsoid LC droplets between 40 and 100 nm size surrounded by a polymer. The whole sample was spatially discretised at a nanometre scale. The LC director inside the LC droplet was estimated by minimising the free total elastic energy in three dimensions. This minimisation deals with the final LC director distribution, and hence with the permittivity tensor as the input of the three-dimensional split-field FDTD scheme. The analysis covered the anchoring strength and the external control voltage applied to the H-PDLC. The diffraction efficiency of an H-PDLC was analysed as a function of the anchoring and the voltage for oblique angle of incidence. The results are consistent and show the potential of this scheme to analyse the behaviour of LC-based devices accurately. The analysis of the influence of both anchoring strength and external control voltage corroborated that the anchoring strength dominates the LC orientation of the droplets in the low–mid range of the external control voltage. For weak anchoring, the LC director was aligned towards the normal direction easily compared to the strongest anchoring scenario. In this specific case, the simulation demonstrated that the surface anchoring in LC droplets influences in a great manner the LC orientation of the whole H-PDLC structure.

It is worth noting that with this approach, not only can direct parameters from the electromagnetic field be obtained, but polarisation information and diffraction efficiencies, amongst others, can also be indirectly computed from the raw SF-FDTD output in a single simulation.

The authors are currently working on considering additional terms in the minimisation of the total free energy, such as the $K_{13}$. Applying this setup with some approximations and machine learning techniques could transform this scheme into a powerful method for characterisation of H-PDLC samples through the inverse analysis of experimental diffraction efficiency curves.

## Figures and Tables

**Table 1:** Parameter values used for numerical simulations.

Parameter Description	Symbol	Value
Frank-Oseen elastic coefficients	$K_{11},K_{22},K_{33},K_{24}$	6.82 pN, 3.9 pN, 5.74 pN, 4.00 pN
refractive indices LC	$n_{e},n_{O}$	1.84, 1.54
refractive index of the polymer	$n_{\mathrm{pol}}$	1.53
relative dielectric permittivity	$\epsilon_{\|},\epsilon_{⊥}$	14, 8.5
thickness of the grating	*d*	8 μm
cell size	Δx=Δy=Δz	12.67 nm
maximum voltage	$\Phi_{\max}$	200 V
Wavelength	$\lambda_{0}$	633 nm
Time step	Δt	$s\Delta x/c\approx 2.11\cdot 10^{-17}s$

It is worth noting that the time resolution depends on the Courant stability factor [25], which is also influenced by the angle of incidence. The value provided in Table 1 is for normal incidence ($\theta_{0}=0^{\circ }$).

## Abbreviations

The following abbreviations are used in this manuscript:

H-PDLC	Holographic polymer dispersed liquid crystal
SF-FDTD	Split field finite-difference time-domain
